# First, do no harm

**DOI:** 10.1080/19420889.2017.1393593

**Published:** 2017-12-04

**Authors:** Thomas E. Marler, Anders J. Lindström

**Affiliations:** a Western Pacific Tropical Research Center, University of Guam, UOG Station, Mangilao, Guam, USA; b Nong Nooch Tropical Botanical Garden, Sattahip, Chonburi, Thailand

**Keywords:** conservation, context dependency, endangered species recovery, restoration ecology

## Abstract

Conservation agencies charged with care of threatened plant species should be governed by the concepts that conservation actions should do no harm. Adaptive management research progresses in imperfect situations due to incomplete knowledge. Interpreting new experimental or observational evidence for inclusion in conservation plans should first consider the big picture by identifying collateral quandaries before scaling up the approach to large-scale implementation. We discuss a case study of *Cycas micronesica* conservation activities on the island of Guam. The use of large stem cuttings has been shown to be a viable approach for rescuing trees from planned construction sites. However, this artificial means of producing transplants exhibits shortcomings, some of which may add new threats to the existing plant population. Moreover, devoting funds for use of the new technique in tree rescue projects does not address the primary threats that have led to listing under the United States Endangered Species Act (ESA). Transplanted trees will likely succumb to those ubiquitous threats shortly after the completion of a successful rescue project. Alternatively, investing conservation funds into mitigation of the primary threats could lead to removal of the species from the ESA.

## Introduction

New knowledge developed from adaptive management research often reveals a protocol that can be added to a conservation goal. But conservation actions need to be remedial to be worthy of implementation, especially if they require large amounts of funding or human resources. Acknowledging all integrative facets of uncertainty connected to a newly available protocol is important to maintain the role of scientific rigor in the conservation efforts. Exploring the newly uncovered uncertainties with transparency will ensure development of the most effective conservation actions. Therefore, a new protocol should not be automatically implemented on a large scale without exploring the unintended consequences.

**Figure 1.**
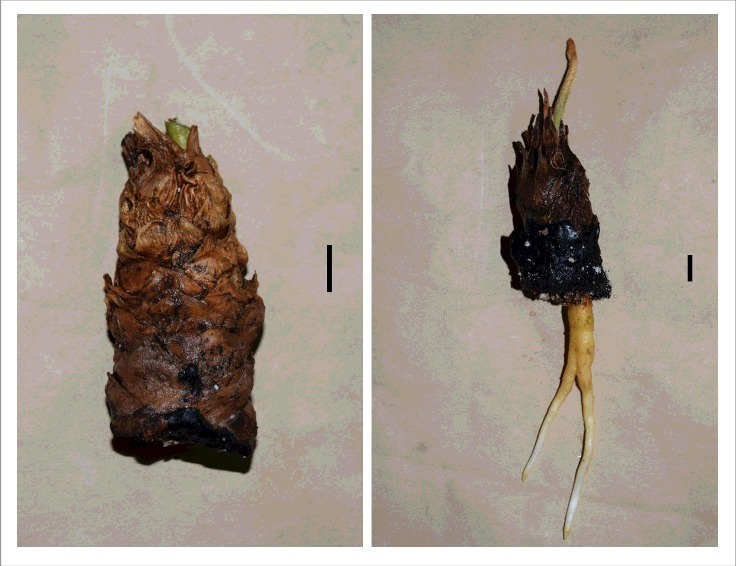
Small stem cuttings (left) have long been used for asexual propagation of *Cycas micronesica* and other cycad species. With appropriate treatment, new root and leaf growth are evident in two months (right). Bars represent 1-cm.

Small lateral stems have long been used for asexual propagation of cycad species (Fig. 1). A recent adaptive management study from the island of Guam has revealed that the same protocols can be successful with large stem sections from the pachycaulous *Cycas micronesica* tree.^1^ The results verified this approach could be employed to conserve some of the resident gene pool from an *in situ* sub-population by creating large transplants from the existing trees. Using the technique would provide conservation agencies with the ability to create transplants while bypassing the expenses that accompany traditional excavation techniques required to remove an intact root ball.

## Collateral issues

Several quandaries have emerged with the new knowledge that large stem cuttings can be used for vegetative propagation of *C. micronesica* trees. We illuminate several of these quandaries.

a. Conservation Premise: Gains in knowledge that hold the potential to improve recovery efforts of threatened plant species must be implemented with contextual dependency.

Guam Case: The results of this study revealed that an experienced cycad horticulturist may expect roughly 40% success in adventitious root formation on large *C. micronesica* stem cuttings. The trees employed for the study were compromised in health due to seven years of infestations by the invasive *Aulacaspis yasumatsui* and two other non-native pests. As a result, the expected outcome of 40% success should not be expected for *C. micronesica* plants subjected to longer than seven years of infestations.

Synopsis: More research is required to unambiguously predict percent success when using trees with pest infestations longer than seven years. Conservation policy makers and project planners should expect less than 40% success when attempting to develop adventitious roots on stems from Guam's contemporary *C. micronesica* population, which has experienced more than a decade of ubiquitous pest pressure.

b. Conservation Premise. Native tree species possess resistance traits that minimize damage by native abiotic and biotic stressors. When artificial conservation protocols are developed for managing the population of a threatened native species, these intrinsic resistance traits may not be retained throughout the newly available contrived protocols.

Guam Case: Tropical cyclones are more frequent on Guam than in any other locality of the United States.^2^ Prior to the non-native insect invasions that have endangered *C. micronesica*, the Guam population experienced minimal root anchorage failure during tropical cyclones^3^ and rapid tree health recovery following tropical cyclones.^4^ Traditional winching techniques were used to predict that sustained *A. yasumatsui* infestations compromise the mechanical properties of *C. micronesica* stems,^5^ and these predictions were confirmed in May 2015 when increased stem failure occurred during Typhoon Dolphin.^6^ However, the sustained pest infestations did not influence the extent of *C. micronesica* root failure during Typhoon Dolphin, indicating the original roots maintained their anchorage traits.

Large stems used to develop adventitious roots are restricted by new root growth that emerges through *de novo* organogenesis from stem tissue. To date, no studies have been enacted on any cycad species to determine if anchorage properties of adventitious roots differ from those of the taproot and lateral systems of zygotic roots. The most commanding published information addressing this premise is from data following Hurricane Andrew in southern Florida.^7^ About one-third of the Tahiti lime (*Citrus* x *latifolia*) trees growing on zygotic root systems were uprooted by the tropical cyclone, but 91% of the trees growing on adventitious root systems were uprooted.

Synopsis: Using all available knowledge, cycad transplants created from large stems with adventitious roots will not retain the native root anchorage traits that provide this species with resistance to tropical cyclone stress. These large trees supported by adventitious roots will be uprooted during the first tropical cyclone that occurs following a *C. micronesica* rescue operation.

c. Conservation Premise: Refinement of a new conservation protocol for threatened tree species may improve resistance to a native stressor, but may decrease resistance to a non-native stressor.

Guam case: We have shown that root formation on large *C. micronesica* stem cuttings is possible for rescue of the gene pool from an *in situ* population.^1^ We predicted an increase in propagation success by use of small cuttings rather than large cuttings. This approach would also create transplants that are small in stature when they are out-planted in the recipient locations, which would reduce the wind drag during a tropical cyclone and decrease the chance of toppling during the years immediately after the transplant operation. Unfortunately, the introduced *Rhyzobius lophanthae* predator does not forage near the forest floor, so the ubiquitous *A. yasumatsui* population is not controlled on *C. micronesica* leaves at lower stratum.^8^ This behavior of the predator is one reason that seedling and juvenile plants were the first to be killed from the cycad populations following the invasion of *A. yasumatsui*.^9^ We have been monitoring *in situ* permanent plots in northern Guam since the *A. yasumatsui* invasion, and the number of *C. micronesica* plants less than 2-m in height has been reduced from 2,698 per ha in 2004 to only 17 plants per ha today (unpublished data). Moreover, all enduring live plants are greater than 1.75-m in height. We use these findings to estimate any transplants created by adventitious rooting of stem cuttings less than 1.75-cm in length would likely be killed by the consortium of alien insect herbivores shortly after a rescue and transplant project.

Synopsis: Adventitious root formation on short *C. micronesica* stem cuttings will stratify the new leaves where the *R. lophanthae* predator does not forage. Transplants created from small stems with adventitious roots will be killed by *A. yasumatsui* infestations shortly after a tree rescue project.

d. Conservation Premise: Translocation projects are used in conservation to expand existing sub-populations and increase the geographic range of an endangered plant species. These approaches effectively conserve some of the donor sub-population. Conservation issues involving the organisms in the recipient location should be fully identified and discussed before proceeding with these actions.

Guam case: Rescuing the genetic breadth of a donor *C. micronesica* sub-population and transplanting that gene pool to a recipient sub-population is now possible with the use of adventitious root formation on large stem cuttings.^1^ But positioning of the recipient translocation site must consider the gene pool of the resident con-specific subpopulation.

Gene flow among Guam's *C. micronesica* sub-populations is minimal.^10^ This conservation knowledge indicates that salvaged trees should not be planted in existing sub-populations at considerable distance from the donor sub-population, as the non-local genes would unnaturally alter the gene pool within the recipient habitat. Other plant species listed under the ESA are also being salvaged on Guam from the same construction sites, and are being prepared for translocation to new recipient sites. This is proceeding without understanding the genetic structure among the sub-populations of those species throughout Guam. A failure to first understand the genetic considerations when native species are used in any form of ecosystem restoration may put at risk the long-term success of the conservation efforts.^11^ Our model with *C. micronesica* should be implemented with these other endangered species before any recipient sites are identified. This would begin with development of adequate markers for use with contemporary techniques^12,13^ and culminate with determination of the amount of gene flow among existing sub-populations.^10^

Synopsis: Large expanses of central Guam are devoid of *in situ C. micronesica* populations. Some of these isolated sites could be identified and used as the recipient sites to accept salvaged *C. micronesica* trees. Alternatively, propagated trees could be returned to habitats that are adjacent to the original donor salvage site. Transplanting cycad trees from one site in order to conserve that gene pool into a second site with existing trees will lead to genetic contamination of the recipient site by non-local genes.

e. Conservation Premise: The primary threats that lead to a tree species becoming listed as threatened or endangered should govern all conservation decisions. Enacting conservation projects that are expensive and fail to address those original threats may be a waste of resources that could be dedicated to mitigate the original threats.

Guam case: The biotic threats that were initiated with the 2003 invasion of *Aulacaspis yasumatsui* led to listing of *C. micronesica* under the Red List of the International Union for Conservation of Nature & Natural Resources^14^ and under the ESA.^15^ These threats persist throughout Guam today, and the salvage projects funded to rescue trees from construction sites do not address those primary threats in any capacity. Trees that are rescued and transplanted will be destined to subsequent mortality by the ubiquitous biotic threats.

*Cycas micronesica* plants have been cultivated for more than 18 years at Nong Nooch Tropical Botanical Garden in Thailand, which is within the native range of *A. yasumatsui*. The plants represent one of 112 *Cycas* species, all of which serve as host for *A. yasumatsui*, which are planted in various co-mingled sites. Under these conditions, the *C. micronesica* plants from Guam, Rota, Palau, and Yap have never exhibited greater susceptibility to *A. yasumatsui* than the other *Cycas* species. The *A. yasumatsui* population is adequately controlled without pesticides in this setting where native biological control of the armored scale population occurs. These direct observations indicate that establishment of adequate biological control on Guam and Rota would eliminate the primary threats to the plant population. Indeed, continued research and implementation of biological control of this armored scale should be pursued.^16^

The scale predator *R. lophanthae* was released in Guam in 2005 and a fortuitous establishment of the scale parasitoid *Arrhenophagus chionaspidis* was observed in 2013. Additionally, numerous introductions and releases of *Coccobius fulvus* and *Aphytis lingnanensis* parasitoids have been conducted on Guam, but to date we have not recorded any evidence that these parasitoids have established. The lethal armored scale population continues to kill the *C. micronesica* trees and does not reveal a pattern that indicates plant mortality will subside in the near future.

The conservation community should invest all available efforts into liaising success in combating this primary threat. Conservation funds should be made available to continue search for more effective biological control. The failures to find new parasitoids that could be introduced to Guam and Rota have not been due to an inability to locate new parasitoids within the native range of *A. yasumatsui*. Instead, the inability to obtain import permits to bring those parasitoid taxa to Guam has been a direct result of our inability to obtain unambiguous taxonomic identification of the collected parasitoids. This is a result of fewer taxonomists available to support our conservation needs today in comparison to historical time periods. The long-term approach to fixing this conservation dilemma would argue for a reversal of the academic trends that are leading to fewer scientists with traditional applied skills such as taxonomy. The short-term approach for Guam would be to fund more collecting trips in hopes of locating parasitoids that can be identified to species level, which would enable immediate application for import permits. Moreover, available funds may urge skilled taxonomists to be willing to prioritize Guam's needs of describing parasitoids that are new to science such that a new binomial becomes available to apply for permits.

Synopsis: *Cycas micronesica* trees rescued during successful transplant projects will eventually die along with the rest of the *in situ* population if the greatest biotic threats remain un-mitigated. Tree rescue projects will ultimately be unsuccessful, and if the conservation funds are instead redirected to expanded biological control efforts, the plant mortality will cease and the species can be removed from the ESA-listing. This would ultimately save vast amounts of government resources because future construction activities could proceed without the need for costly plant rescue projects.

## Summation

Major concepts in medical ethics are embodied in the Latin phrase *primum non nocere* (first, do no harm). Conservation agencies charged with care of threatened plant species should be governed by these same concepts. Ensuring that no action is implemented that may harm the plants in a conservation project is arguably more important than executing an action that may be beneficial for their health.

We have argued that the role of research in conservation efforts is often lacking, and if a portion of conservation funds are devoted to research the overall conservation goals will be met with more success.^17^ But adaptive management research progresses in imperfect situations due to incomplete knowledge. Therefore, interpreting the results then implementing those results into conservation plans must embrace an intellectual approach. Our case study^1^ provides an example of how a layer of knowledge can be added that enables a successful approach for conserving the gene pool of a tree species from a donor subpopulation, making it available for transplanting to a new sub-population. However, decision-makers should pause to consider the big picture by addressing collateral quandaries before scaling up the approach to large-scale implementation.

*Primum non nocere*, large *C. micronesica* stems with adventitious roots will likely experience root anchorage failure during the first tropical cyclone following a Guam salvage project, as the anchorage traits of adventitious roots are inferior. But trees left intact would not exhibit root anchorage failure. *Primum non nocere*, small stems with adventitious roots will likely be killed by the ubiquitous *A. yasumatsui* pressure throughout Guam, as the armored scale is not controlled in the lower stratum by the established predator *R. lophanthae*. But the large trees left intact would remain alive much longer as they have no leaves in the lower stratum. *Primum non nocere*, moving *C. micronesica* genes from one sub-population to a second sub-population will threaten the unique gene pool of the recipient Guam sub-population. But trees left intact would not damage the genetic integrity of any sub-population. *Primum non nocere*, copious levels of funding to rescue existing *C. micronesica* trees from future constructions sites on Guam fail to address the primary threat that endangered the tree species. But redirecting those funds toward expanded biological control of *A. yasumatsui* would mitigate the threats and enable delisting of the species. This would also eliminate the need to spend conservation funds for continued tree rescue projects.

## References

[1] Marler TE, Cruz GN Adventitious rooting of mature *Cycas* *micronesica* K.D. Hill tree stems reveals moderate success for salvage of an endangered cycad. J Threatened Taxa. 2017;9:10565–70. doi:10.11609/jott.3523.9.8.10565-10570.

[2] Marler TE Tropical cyclones and perennial species in the Mariana Islands. HortScience. 2001;36:264–8.

[3] Marler TE, Hirsh H Guam's *Cycas* *micronesica* population ravaged by Supertyphoon Paka. HortScience. 1998;33:1116–8.

[4] Hirsh H, Marler T Damage and recovery of *Cycas* *micronesica* after Typhoon Paka. Biotropica. 2002;34:598–602. doi:10.1111/j.1744-7429.2002.tb00579.x.

[5] Marler TE Increased threat of island endemic tree's extirpation via invasion-induced decline of intrinsic resistance to recurring tropical cyclones. Commun Integr Biol. 2013;6:e22361. doi:10.4161/cib.22361. 23802037 PMC3689569

[6] Marler TE, Lawrence JH, Cruz GN Topographic relief, wind direction, and conservation management decisions influence *Cycas* *micronesica* K.D. Hill population damage during tropical cyclone. J Geogr Nat Disasters. 2016;6:3. doi:10.4172/2167-0587.1000178.

[7] Crane JJ, Schaffer B, Campbell RJ Recovery from hurricanes and the long-term impacts on perennial tropical fruit crops in South Florida. HortScience. 2001;36:258–63.

[8] Marler TE, Miller R, Moore A Vertical stratification of predation on *Aulacaspis* *yasumatsui* infesting *Cycas* *micronesica* seedlings. HortScience. 2013;48:60–2.

[9] Marler TE, Lawrence JH Demography of *Cycas* *micronesica* on Guam following introduction of the armoured scale *Aulacaspis* *yasumatsui*. J Trop Ecol. 2012;28:233–42. doi:10.1017/S0266467412000119.

[10] Cibrián-Jaramillo A, Daly AC, Brenner E, DeSalle R, Marler TE When North and South don't mix: genetic connectivity of a recently endangered oceanic cycad, *Cycas* *micronesica*, in Guam using EST-microsatellites. Mol Ecol. 2010;19:2364–79. PMID:20497328. 20497328 10.1111/j.1365-294X.2010.04638.x

[11] Bozzano M, Jalonen R, Thomas E, Boshier D, Gallo L, Cavers S, Bordács S, Smith P, Loo J, eds. Genetic considerations in ecosystem restoration using native tree species. State of the World's Forest Genetic Resources – Thematic Study. Rome. FAO and Bioversity International; 2014 p. 281.

[12] Cibrián-Jaramillo A, Marler TE, DeSalle R, Brenner ED Development of EST-microsatellites from the cycad *Cycas* *rumphii*, and their use in the recently endangered *Cycas* *micronesica*. Conserv Genomics. 2008;9:1051–4. doi:10.1007/s10592-007-9447-3.

[13] Cibrián-Jaramillo A, Marler TE Novel tools for an old lineage. Commun Integr Biol. 2011;4:466–8. doi:10.4161/cib.15546. 21966573 PMC3181523

[14] Marler T, Haynes J, Lindström A *Cycas* *micronesica*. In: IUCN 2014. IUCN Red List of Threatened Species. Version 2014.2. Available at: http://www.iucnredlist.org. Downloaded on 6 Oct 2017.

[15] United States Fish & Wildlife Service Endangered and threatened wildlife and plants; endangered status for 16 species and threatened status for 7 species in Micronesia. Fed Regist. 2015;80:59424–97.

[16] Tang W, Cave RD Recent advances in the biological control of cycad aulacaspis scale. Encephalartos. 2016;123:16–18.

[17] Marler TE, Lindström AJ The value of research to selling the conservation of threatened species: the case of *Cycas* *micronesica*. J Threatened Taxa. 2014;6:6523–8. doi:10.11609/JoTT.o4098.6523-8.

